# The Use of F-18 FDG PET-Based Cognitive Reserve to Evaluate Cognitive Decline in Alzheimer’s Disease, Independent of Educational Influence

**DOI:** 10.3390/medicina59050945

**Published:** 2023-05-14

**Authors:** Hyung Jin Choi, Minjung Seo, Ahro Kim, Seol Hoon Park

**Affiliations:** 1 Department of Nuclear Medicine, Ulsan University Hospital, Ulsan 44033, Republic of Korea; drchoihj@uuh.ulsan.kr; 2 Department of Nuclear Medicine, University of Ulsan College of Medicine, Ulsan University Hospital, Ulsan 44033, Republic of Korea; 0733285@uuh.ulsan.kr; 3 Department of Neurology, University of Ulsan College of Medicine, Ulsan University Hospital, Ulsan 44033, Republic of Korea

**Keywords:** Alzheimer’s disease, brain function reserve, cognitive reserve, dementia, FDG, PET

## Abstract

*Background and Objectives*: The optimal assessment of cognitive function, including the impact of education, is crucial in managing Alzheimer’s disease (AD). This study aimed to evaluate the role of cognitive reserve (CR), represented by the metabolic status of regions of the cerebral cortex, to evaluate cognitive decline considering the educational attainment of patients with AD. *Materials and Methods:* We used data from the Alzheimer’s Disease Neuroimaging Initiative database, and selected 124 patients who underwent both baseline F-18 fluorodeoxyglucose (FDG) and F-18 florbetaben (FBB) positron emission tomography (PET) scans. Demographics, cognitive function variables (Clinical Dementia Rating—Sum of Boxes [CDR]; AD Assessment Scale 11/13 [ADAS11/13] Mini-Mental State Examination [MMSE]), and the average standardized uptake value ratio (SUVR) of cerebral cortex regions to those of the cerebellum were obtained from the data. The participants’ education level was divided into low and high education subgroups using four cut-offs of 12, 14, 16, and 18 years of educational attainment (G12, G14, G16, and G18, respectively). Demographic and cognitive function variables were compared between the two subgroups in each of the four groups, and their correlations with the SUVRs were evaluated. *Results:* There was no significant difference between the high and low education subgroups in each of the four groups, except for ADAS11/13 and MMSE in G14 and age in G16. The SUVRs of FDG PET (FDG_SUVR_) were significantly correlated with CDR, ADAS11/13, and MMSE scores. FDG_SUVR_ showed different trajectories of neurodegeneration between the low and high education groups. *Conclusions:* FDG_SUVR_ correlated moderately but significantly with neuropsychological test results, without being influenced by education level. Therefore, FDG PET may reflect CR independent of education level, and therefore could be a reliable tool to evaluate cognitive decline in AD.

## 1. Background

Alzheimer’s disease (AD) is a neurodegenerative disorder characterized by the mismatched production and clearance of β-amyloid (Aβ) and tau proteins. This is followed by adverse neuro-inflammation. Cognitive decline is the most common symptom of AD, and is a key factor in the diagnosis and evaluation of AD progression and treatment response. Conventional neuropsychological tools used to measure cognitive function include the Mini-Mental State Examination (MMSE), the Clinical Dementia Rating (CDR), and the AD Assessment Scale (ADAS) [1,2,3].

The discrepancy between the severity of cognitive decline and postmortem pathological findings among participants in the low and high education groups has been termed “cognitive reserve (CR)” [4,5]. The CR signifies the influence of education in the assessment of cognitive function. The MMSE, which is a representative cognitive test, may also be influenced by the education level of individuals [6]. Therefore, an appropriate measurement tool is required to assess cognitive decline, particularly for cases in which the education level of the patient may have impacted the optimal assessment of cognitive function [4].

F-18 F-18 fluorodeoxyglucose (FDG) positron emission tomography (PET) is used as an image biomarker to assess AD, and for the differential diagnosis of other dementia types. Amyloid PET depicts the accumulation of Aβ and plays a role in the diagnosis of AD [7,8,9]. The correlation of cognitive function with FDG and amyloid PET has been extensively evaluated [10,11,12]. The standardized uptake value ratio (SUVR) of FDG PET, using the cerebellum as a reference, is used as a marker of cognitive decline [11] and may more accurately represent the CR without the interference of education level, thereby enabling the optimal assessment of cognitive function [4].

Therefore, this study aimed to evaluate the correlation of FDG PET-based SUVR with neuropsychological test results according to education level and to assess whether the measurement of SUVR by FDG PET can be a surrogate for conventional cognitive measures and a proxy for education-independent CR in AD.

## 2. Materials and Methods

### 2.1. Dataset from the Alzheimer’s Disease Neuroimaging Initiative

Several key variables from various clinical information reports and biomarker results from the Alzheimer’s Disease Neuroimaging Initiative (ADNI) protocols are merged to create the “adnimerge” table. A total of 2159 participants in ADNI-3 datasets were screened and 124 participants who had both baseline FDG PET and florbetaben (FBB) PET amyloid imaging were finally enrolled. The FDG_SUVR_ and FBB_SUVR_ of 124 participants were analyzed in this study. The FDG_SUVR_ was defined as the average of the FDG PET-based SUVR values of the angular, temporal, and posterior cingulate regions. The FBB_SUVR_ was defined as the average of the FBB PET-based SUVR values of the frontal cortex, anterior cingulate gyrus, precuneus cortex, and parietal cortex. The educational attainment in years (EDU), number of apolipoprotein E4 alleles (APOE), MMSE score, CDR, and ADAS11/13 data were also obtained from the baseline study of ADNI datasets. The APOE results were available for 99 of the 124 (79.8%) participants. Cerebrospinal fluid Aβ data were not available. The Institutional Ethics Committee of Ulsan University Hospital confirmed that ethical approval was not required for this observational study, and waived the requirement for informed consent (IRB file number: UUH2022-05-029).

### 2.2. Statistical Analyses

All data are described as mean and standard deviation for continuous variables and as numbers (percentages) for categorical variables. Age, sex, EDU, APOE, CDR, ADAS11/13, MMSE, FDG_SUVR_, and FBB_SUVR_ were compared between mild cognitive impairment (MCI) and AD groups using an independent *t*-test and a Mann–Whitney U test for continuous and categorical variables, respectively. Four cut-offs of 12, 14, 16, and 18 years for EDU were used and participants were divided into low and high education subgroups (G12: ≤12 vs. >12, G14: ≤14 vs. >14, G16: ≤16 vs. >16, and G18: ≤18 vs. >18, respectively). The association of each subgroup of G12, G14, G16, and G18 with neuropsychological results was evaluated using Pearson’s and Spearman’s correlation analyses. For the MCI and AD groups, univariable and multivariable analyses were performed to evaluate the association of age, sex, EDU, APOE, FDG_SUVR_, and FBB_SUVR_ with neuropsychological tests to assess cognitive function. In multivariable regression analyses, dummy variables were used for categorical variables, including sex and APOE.

## 3. Results

### 3.1. Baseline Demographics

There were no statistically significant differences regarding age, sex, and EDU between the MCI and AD groups; however, APOE, CDR, ADAS11/13, and MMSE scores, as well as FDG_SUVR_ and FBB_SUVR_, showed significant differences. One of the three healthy participants was 90 years old and had CDR, ADAS11, ADAS13, and MMSE scores of 7, 29.33, 41.33, and 20, respectively. The other two healthy participants exhibited normal neuropsychological results. Detailed demographics of all participants are presented in Table 1.

### 3.2. Differences in Variables between the Low and High Education Subgroups Using Four Cut-Offs for EDU

There were no significant differences in variables between the low and high education subgroups of each of the four EDU groups (G12, G14, G16, and G18), except for the ADAS11 and 13 scores (*p* = 0.038 and 0.032, respectively) and the MMSE score (*p* = 0.017) in G14 (Table 2). However, the low and high education subgroups in the MCI group possessed significantly different MMSE scores (*p* = 0.032 and 0.023, respectively) in G12 and G14, and age and MMSE scores (*p* = 0.012 and *p* = 0.006, respectively) in G16. In the AD group, only ADAS11 and FBB_SUVR_ (*p* = 0.037 and 0.021, respectively) in G16 exhibited statistically significant differences between the low and high education groups (Supplementary Tables S1 and S2).

### 3.3. Correlation between Demographics, FDG_SUVR_, FBB_SUVR_, and Neuropsychological Tests

Correlation analyses for all participants revealed that APOE, FDG_SUVR_, and FBB_SUVR_ were correlated with CDR, ADAS11/13, and MMSE scores. Age correlated with ADAS11/13; however, the correlation was not statistically significant (Table 3). In the MCI group, similar correlation patterns for FDG_SUVR_, FBB_SUVR_, and age with CDR, ADAS11/13, and MMSE were observed. The AD group showed almost no correlations with the results of the neuropsychological tests (Supplementary Tables S3 and S4).

### 3.4. Multivariable Analyses for Correlations between Demographics, FDG_SUVR_, FBB_SUVR_, and Neuropsychological Tests

**Figure 1:**
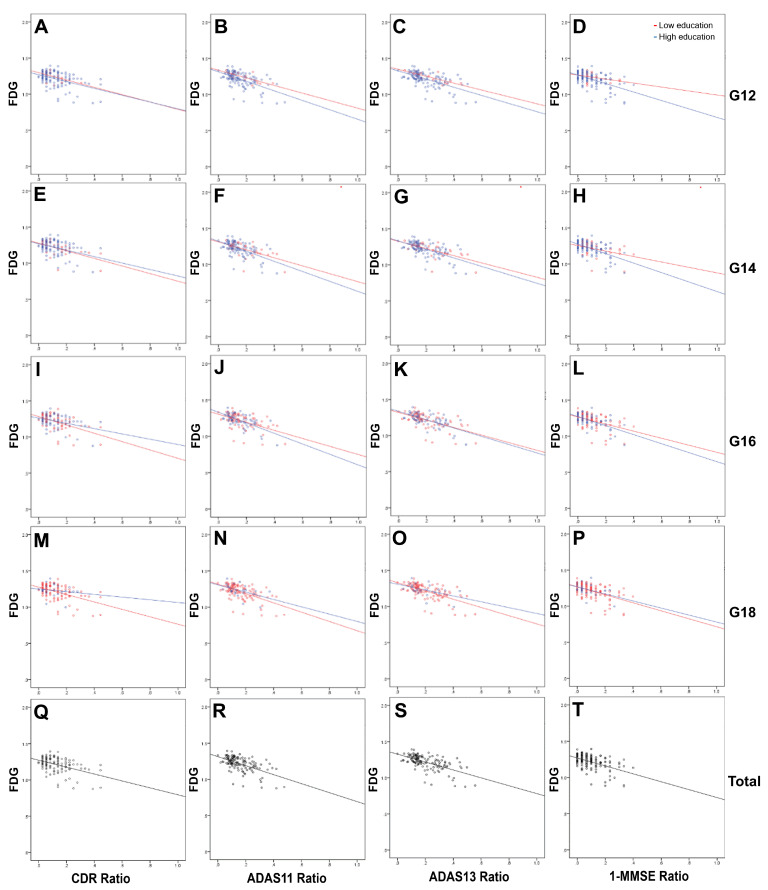
Linear regression analyses of FDG_SUVR_ with neuropsychological results for all participants. Regression plots and lines between the low- and high-education subgroups were created for G12 (**A**–**D**), G14 (**E**–**H**), G16 (**I**–**L**), G18 (**M**–**P**), and total participants (**Q**–**T**). The CDR, ADAS11/13, and MMSE scores were divided by the maximum value for each test (18, 70, 85, and 30, respectively) and then normalized from 0 to 1 to facilitate equivalent visual comparisons. For the same reason, 1—MMSE was used for all graphs.

**Figure 2:**
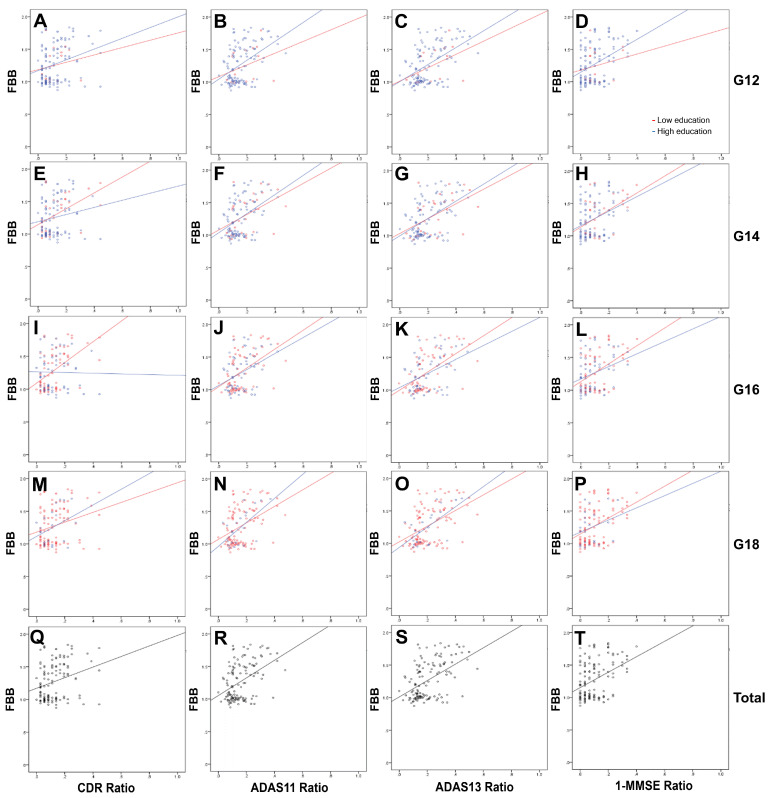
Linear regression analyses of FBB_SUVR_ with neuropsychological results for all participants. Regression plots and lines between the low- and high- education subgroups were created for G12 (**A**–**D**), G14 (**E**–**H**), G16 (**I**–**L**), G18 (**M**–**P**), and total participants (**Q**–**T**). The CDR, ADAS11/13, and MMSE scores were divided by the maximum value for each test (18, 70, 85, and 30, respectively) and then normalized from 0 to 1 to facilitate equivalent visual comparisons. For the same reason, 1—MMSE was used for all graphs.

Multivariable linear regression analyses revealed that FDG_SUVR_ was a significant independent factor correlating with CDR, ADAS11/13, and MMSE in most groups, followed by FBB_SUVR_ and age. Sex, EDU, and APOE were not significant variables (Table 4). The regression lines of FDG_SUVR_ and FBB_SUVR_ for CDR, ADAS11/13, and MMSE scores are shown in Figure 1 and Figure 2. The FDG_SUVR_ downward slopes for the participants with high education levels for the ADAS11/13 and MMSE scores in the G12, G14, and G16 groups were steeper than those for the participants with low education levels. The slopes for CDR in all EDU groups as well as the ADAS11/13 and MMSE scores in the G12, G14, and G16 groups were gentler for those with high than for those with low education levels (Figure 1). The upward sloping lines of FBB_SUVR_ were not as regular as the downward sloping lines of FDG_SUVR_ (Figure 2).

## 4. Discussion

With the global aging of society, AD is increasingly becoming a public health issue [13]. Cognitive decline becomes more severe over time, because of the progressive neurodegenerative nature of AD. However, education has demonstrated a protective influence against AD [14,15], which led to the hypothesis that CR might cause the delayed detection of AD or its progression, particularly in highly educated populations [4]. Therefore, we evaluated whether FDG_SUVR_ correlated with the results of various neuropsychological tests used to evaluate CR, and whether FDG_SUVR_ could reflect CR in a manner that was independent of the influence of education. We observed that FDG_SUVR_ was significantly and moderately correlated with CDR, ADAS11/13, and MMSE scores.

In our study, the age of high-education-level participants at baseline was expected to be older than that of the low-education-level participants, as their neuropsychological results did not differ according to the concept of CR. However, we found that the age at baseline did not differ between patients with low or high education levels. In contrast, the ADAS11/13 and MMSE differed for all participants in G14, and the MMSE scores for the MCI group differed in G12 and G14 between the low- and high-education groups. Only in the MCI group for G16 did age differ significantly according to different MMSE results. None of the other groups exhibited significant differences. These results suggest that CR may not affect the early detection of AD, regardless of the education level.

Therefore, the question might arise regarding whether the concept of CR is meaningful. Since CR could delay AD diagnosis in the higher education group, FDG and amyloid PET were evaluated as tools to assess CR, regardless of the level of education [4,5,15]. Interestingly, FDG_SUVR_ and FBB_SUVR_ revealed a moderate correlation with neuropsychological test results across all EDU-based subgroups in our study. Regression lines of FDG_SUVR_ and FBB_SUVR_ (Figure 1 and Figure 2) data at baseline showed no significant differences between the low- and high-education level groups (left side of the lines), in which neuropsychological results ranged in severity from mild to moderate. However, more distinctive differences might be observed if the lines were extended to more severe neuropsychological results on the right side. The virtual differences in FDG_SUVR_ and FBB_SUVR_ between the low- and high-education level groups might indicate the existence of CR, although this was not based on an analysis with real data. In addition, longitudinal correlations between FDG or FBB PET/CT and ADAS11 have been presented in other studies [5,16]. Another study using C-11 Pittsburgh Compound B (PiB) reported no relationship between education and CR in participants with lower PiB uptake, which might represent the early stage of AD pathology, similar to the left side of the regression lines in our study. In addition, the PiB study highlighted that the duration of education was correlated with the CDR and MMSE scores in participants with higher PiB uptake, which might reflect the advanced pathological changes of AD (similar to the right side of the lines in our study) [17]. FDG and amyloid PET imaging are representative biomarkers of pathology and neurodegeneration in AD [9], thereby suggesting that CR might explain the different neurodegenerative trajectories between low- and high-education-level groups. The gap in the trajectories in terms of subjective or objective cognitive decline between low- and high-education-level groups might be minimal at the time of the initial workup, but may become more pronounced as cognitive impairment progresses. Therefore, CR might play a more important role in evaluating treatment responses than in the diagnosis of AD.

Neuropsychological tests remain the mainstay for evaluating treatment responses in patients with AD. Conducting neuropsychological tests in an AD population with profound cognitive decline is challenging. Questionnaire-based neuropsychological assessments may be difficult to obtain, particularly for individuals with intellectual disabilities. Indeed, the low sensitivity of questionnaire-type cognitive assessments has been previously reported [18]. The evaluation of cognitive function using neuropsychological assessments, including the MMSE, remains controversial [19,20]. Other studies have indicated that the educational level of participants might affect the MMSE results [21,22]. Our study revealed that the regression lines of FDG_SUVR_ and FBB_SUVR_ were more reliable in representing CR with the current cognitive status demonstrated by neuropsychological test results, independent of educational levels. In multivariable analyses, FDG_SUVR_ was the factor most significantly correlated with CDR, ADAS11/13, and MMSE scores, followed by FBB_SUVR_ and age. Other studies also showed that FDG_SUVR_ and FBB_SUVR_ were significantly correlated with cognition [23]. In addition, those studies hypothesized that the correlation of FDG_SUVR_ with cognitive status may be more significant than that of FBB_SUVR_, as our study also indicated [16,23,24]. Similarly, a previous study reported that FDG and amyloid PET/CT might be useful tools to evaluate the concept of CR in mild cases of AD [5,10]. These findings suggest that FDG PET could be useful as a surrogate for the evaluation of cognitive decline, considering the concept of CR, not only for diagnosis but also for treatment response.

Our study had some limitations. This was a cross-sectional study, in which chronological changes in cognitive decline were not monitored. The duration of subjective or objective cognitive impairment could vary and affect the time-based correlation between FDG_SUVR_ and neuropsychological results, which were not evaluated in this study. However, neuropsychological assessments become more difficult as cognitive decline progresses. Nevertheless, our study indicated that even in advanced cases FDG_SUVR_ could provide reliable information on CR, which showed different trajectories between low and high education level participants. Our study was further limited in that only the average FDG_SUVR_ of the angular, temporal, and posterior cingulate regions in the brain were included. As regional changes in the brain glucose metabolism in AD have been well evaluated, more detailed correlations with each brain region based on time and neuropsychological results might provide a better understanding of CR. Further studies should be designed based on these considerations.

## 5. Conclusions

In conclusion, FDG_SUVR_ showed significant correlations with the results of neuropsychological tests based on questionnaires. The FDG_SUVR_ displayed different trajectories of neurodegeneration between participants with low and high education levels based on diminished cognitive function, supporting the concept of CR. Therefore, FDG PET could be a reliable and objective imaging tool to assess cognitive decline and might be a proxy of CR which is independent of educational influence in patients with AD.

## Figures and Tables

**Table 1:** Baseline demographics of all participants.

	Normal	MCI	AD	Total
No.	3	91	30	124
Age, years	79.4 ± 15.9	71.8 ± 7.9	73.9 ± 7.8	72.5 ± 8.0.
Sex, n (%)				
Male	2 (67)	51 (56)	19 (63)	72 (58)
Female	1 (33)	40 (44)	11 (37)	52 (42)
EDU	17.7 ± 2.1	16.3 ± 2.5	16.2 ± 2.5	16.3 ± 2.5
APOE, n * (%)				
0	2 (100)	37 (54)	4 (14)	43 (43)
1	0 (0)	26 (38)	15 (54)	41 (41)
2	0 (0)	6 (8)	9 (32)	15 (16)
CDR *	2.3 ± 4.0	1.6 ± 1.0	4.1 ± 1.8	2.2 ± 1.7
ADAS11 *	13.9 ± 13.9	8.7 ± 3.7	19.2 ± 5.5	11.4 ± 6.4
ADAS13 *	19.6 ± 19.6	13.7 ± 5.7	29.8 ± 7.1	17.8 ± 9.5
MMSE *	25.3 ± 4.7	28.1 ± 7.5	23.8 ± 2.8	27.0 ± 2.8
FDG_SUVR_ *	1.118 ± 0.210	1.243 ± 0.077	1.135 ± 0.111	1.214 ± 0.102
FBB_SUVR_ *	1.302 ± 0.295	1.200 ± 0.252	1.482 ± 0.275	1.271 ± 0.283

Abbreviations: No., number; MCI, mild cognitive impairment; AD, Alzheimer’s disease; EDU, educational attainment in years; APOE, number of apolipoprotein E4 alleles; CDR, clinical dementia rating—sum of boxes; ADAS11/13, AD assessment scale 11/13; MMSE, mini-mental state examination; FDG_SUVR_, average standardized uptake value ratio (SUVR) of angular, temporal, and posterior cingulate in F-18 fluorodeoxyglucose positron emission tomography (PET); FBB_SUVR_, average SUVR of frontal cortex, anterior cingulate, precuneus cortex, and parietal cortex in F-18 florbetaben PET. * (*p* ≤ 0.05) indicates statistically significant differences between the MCI and AD groups only.

**Table 2:** Demographics of the four groups (G12, G14, G16, and G18) based on the educational attainment in years.

EDU	G12		G14		G16		G18	
	≤12	>12	≤24	>14	≤46	>16	≤68	>18
No.	15	109	31	93	67	57	102	22
Age	70.0 ± 5.6	72.8 ± 8.2	70.6 ± 7.6	73.1 ± 8.1	70.4 ± 8.3 *	74.9 ± 6.9 *	71.9 ± 7.9	75.3 ± 8.3
Sex, n (%)								
Male	9 (60)	63 (58)	16 (52)	56 (60)	34 (51)	38 (67)	57 (56)	15 (68)
Female	6 (40)	46 (42)	15 (48)	37 (40)	33 (49)	19 (33)	45 (44)	7 (32)
Diagnosis, n (%)								
AD	3 (20)	27 (25)	10 (32)	20 (22)	15 (22)	15 (26)	26 (25)	4 (18)
MCI	12 (80)	79 (72)	21 (68)	70 (75)	51 (76)	40 (70)	74 (73)	17 (77)
Normal	0 (0)	3 (3)	0 (0)	3 (3)	1 (2)	2 (4)	2 (2)	1 (5)
APOE, n ^†^ (%)								
0	6 (40)	37 (34)	12 (39)	31 (33)	26 (39)	17 (30)	34 (33)	9 (41)
1	3 (20)	38 (35)	10 (32)	31 (33)	23 (34)	18 (31)	39 (38)	2 (9)
2	3 (20)	12 (11)	6 (20)	9 (10)	9 (13)	6 (11)	11 (11)	4 (18)
CDR	2.2 ± 1.4	2.2 ± 1.8	2.5 ± 2.1	2.1 ± 1.6	2.2 ± 1.7	2.3 ± 1.8	2.3 ± 1.8	2.0 ± 1.4
ADAS11	13.2 ± 6.8	11.1 ± 6.4	13.8 ± 7.7 *	10.6 ± 5.7 *	11.6 ± 6.8	11.1 ± 6.0	11.5 ± 6.5	10.9 ± 6.1
ADAS13	19.8 ± 9.9	17.5 ± 9.4	20.9 ± 11.1 *	16.7 ± 8.7 *	17.8 ± 9.8	17.7 ± 9.1	18.0 ± 9.6	17.0 ± 9.1
MMSE	26.5 ± 2.6	27.1 ± 2.8	25.9 ± 3.3 *	27.3 ± 2.6 *	26.7 ± 2.9	27.3 ± 2.8	26.8 ± 2.9	27.6 ± 2.4
FDG_SUVR_	1.236 ± 0.069	1.211 ± 0.105	1.204 ± 0.105	1.217 ± 0.101	1.213 ± 0.108	1.215 ± 0.095	1.211 ± 0.104	1.229 ± 0.089
FBB_SUVR_	1.253 ± 0.269	1.273 ± 0.286	1.314 ± 0.293	1.256 ± 0.280	1.281 ± 0.295	1.259 ± 0.271	1.275 ± 0.289	1.249 ± 0.261

Abbreviations: No., number; G12–18, groups using cut-offs of 12, 14, 16, and 18 years for educational attainment, respectively; EDU, educational attainment in years; AD, Alzheimer’s disease, MCI, mild cognitive impairment; APOE, number of apolipoprotein E4 alleles; CDR, clinical dementia rating—sum of boxes; ADAS11/13, AD assessment scale 11/13; *MMSE*, mini-mental state examination; FDG_SUVR_, average standardized uptake value ratio (SUVR) of angular, temporal, and posterior cingulate in F-18 fluorodeoxyglucose positron emission tomography (PET); FBB_SUVR_, average SUVR of frontal cortex, anterior cingulate, precuneus cortex, and parietal cortex in F-18 florbetaben PET. * (*p* ≤ 0.05) indicates statistical significance. ^†^ APOE results were available for 79.8% of the participants.

**Table 3:** Correlation analyses for demographics, FDG_SUVR_, FBB_SUVR_, and neuropsychological tests.

CDR (Correlation Coefficient)									
	G12		G14		G16		G18		Total
	≤12	>12	≤14	>14	≤16	>16	≤18	>18	
Age ^†^	−0.374	0.178	−0.006	0.225 *	0.120	0.153	0.156	0.116	0.142
Sex ^‡^	−0.016	−0.129	0.069	−0.188	0.037	−0.300^*^	−0.086	−0.295	−0.117
EDU ^†^	−0.066	−0.076	0.132	0.019	−0.129	−0.181	−0.028	0.254	−0.047
APOE ^‡^	0.358	0.290 *	0.446 *	0.226	0.406 *	0.118	0.252 *	0.436	0.290 *
FDG_SUVR_^†^	−0.570 *	−0.444 *	−0.586 *	−0.391 *	−0.514 *	−0.373 *	−0.489 *	−0.163	−0.451 *
FBB_SUVR_^†^	0.164	0.286 *	0.489 *	0.172	0.513 *	−0.019	0.262 *	0.359	0.275 *
ADAS11 (Correlation Coefficient)									
	G12		G14		G16		G18		Total
	≤12	>12	≤14	>14	≤16	>16	≤18	>18	
Age ^†^	−0.311	0.255 *	−0.041	0.343 *	0.092	0.392 *	0.194	0.193	0.189 *
Sex ^‡^	0.000	−0.164	−0.029	−0.210 *	−0.098	−0.272 *	−0.157	−0.177	−0.159
EDU ^†^	−0.018	−0.120	0.068	0.042	−0.269 *	−0.110	−0.191	0.128	−0.155
APOE ^‡^	0.330	0.319 *	0.309	0.332 *	0.342 *	0.292	0.300 *	0.434	0.327 *
FDG_SUVR_^†^	−0.737 *	−0.565 *	−0.578 *	−0.561 *	−0.496 *	−0.653 *	−0.570 *	−0.491 *	−0.559 *
FBB_SUVR_^†^	0.325	0.462 *	0.450 *	0.431 *	0.477 *	0.385 *	0.409 *	0.612 *	0.440 *
ADAS13 (Correlation Coefficient)									
	G12		G14		G16		G18		Total
	≤12	>12	≤14	>14	≤16	>16	≤18	>18	
Age ^†^	−0.230	0.283 *	0.015	0.359 *	0.139	0.381 *	0.230 *	0.237	0.225 *
Sex ^‡^	0.063	−0.130	0.058	−0.188	−0.056	−0.224	−0.120	−0.131	−0.114
EDU ^†^	0.032	−0.116	0.089	0.037	−0.263 *	−0.161	−0.155	0.061	−0.133
APOE ^‡^	0.467	0.349 *	0.379 *	0.350 *	0.425 *	0.287	0.336 *	0.422	0.366 *
FDG_SUVR_^†^	−0.806 *	−0.598 *	−0.622 *	−0.596 *	−0.564 *	−0.649 *	−0.617 *	−0.487 *	−0.599 *
FBB_SUVR_^†^	0.445	0.511 *	0.510 *	0.490 *	0.554 *	0.425 *	0.474 *	0.639 *	0.499 *
MMSE (Correlation Coefficient)									
	G12		G14		G16		G18		Total
	≤12	>12	≤14	>14	≤16	>16	≤18	>18	
Age ^†^	0.404	−0.109	−0.028	−0.120	−0.060	−0.142	−0.062	−0.172	−0.062
Sex ^‡^	0.100	0.018	0.022	0.041	−0.045	0.137	−0.014	0.267	0.023
EDU ^†^	−0.162	0.185	−0.183	0.042	0.184	0.109	0.165	−0.417	0.171
APOE ^‡^	0.095	−0.300 *	−0.179	−0.296 *	−0.299 *	−0.237	−0.239 *	−0.339	−0.268 *
FDG_SUVR_^†^	0.348	0.527 *	0.395 *	0.555 *	0.434 *	0.603 *	0.510 *	0.435 *	0.503 *
FBB_SUVR_^†^	−0.201	−0.418 *	−0.494 *	−0.340 *	−0.444 *	−0.325 *	−0.409 *	−0.287	−0.394 *

Abbreviations: CDR, clinical dementia rating—sum of boxes; G12–18, groups using cut-offs of 12, 14, 16, and 18 years for educational attainment, respectively; EDU, educational attainment in years; APOE, number of apolipoprotein E4 alleles; FDG_SUVR_, average standardized uptake value ratio (SUVR) of angular, temporal, and posterior cingulate in F-18 fluorodeoxyglucose positron emission tomography (PET); FBB_SUVR_, average SUVR of frontal cortex, anterior cingulate, precuneus cortex, and parietal cortex in F-18 florbetaben PET; ADAS11/13, AD assessment scale 11/13; MMSE, mini-mental state examination. * (*p* ≤ 0.05) indicates statistical significance. ^†^ Pearson correlation analysis was used. ^‡^ Spearman correlation analysis was used.

**Table 4:** Multivariable regression analyses between demographics, FDG_SUVR_, FBB_SUVR_, and neuropsychological tests.

CDR (*p*-Value)										
		G12		G14		G16		G18		Total
Mode	EDU	≤12	>12	≤14	>14	≤16	>16	≤18	>18	
Enter										
	Age	0.155	0.044 *	0.552	0.059	0.401	0.300	0.108	0.372	0.089
	Sex (dummy)	0.976	0.868	0.419	0.460	0.321	0.130	0.682	0.525	0.873
	EDU	0.747	0.598	0.406	0.820	0.317	0.072	0.628	0.362	0.573
	APOE (dummy1)	0.838	0.738	0.452	0.630	0.899	0.653	0.946	0.535	0.891
	APOE (dummy2)	0.416	0.097	0.419	0.182	0.630	0.166	0.466	0.188	0.164
	FDG_SUVR_	0.119	<0.001 *	0.012 *	0.002 *	0.002 *	0.018 *	<0.001 *	0.767	<0.001 *
	FBB_SUVR_	0.846	0.847	0.044 *	0.597	0.063	0.152	0.817	0.370	0.853
R^2^		0.578	0.257	0.473	0.215	0.387	0.273	0.264	0.464	0.242
Stepwise ^†^										
	FDG_SUVR_	0.027	<0.001	0.001	<0.001	0.003	0.005	<0.001	-	<0.001
	FBB_SUVR_	-	-	-	-	0.003	-	-	-	-
	Age	-	0.048	-	0.045	-	-	-	-	-
R^2^		0.325	0.227	0.343	0.190	0.360	0.138	0.239	-	0.204
ADAS11 (*p*-value)										
		G12		G14		G16		G18		Total
Mode	EDU	≤12	>12	≤14	>14	≤16	>16	≤18	>18	
Enter										
	Age	0.623	0.007 *	0.498	0.004^*^	0.536	0.005 *	0.034 *	0.271	0.015 *
	Sex (dummy)	0.875	0.995	0.530	0.854	0.555	0.547	0.648	0.565	0.988
	EDU	0.676	0.436	0.363	0.658	0.023 *	0.992	0.012 *	0.515	0.040 *
	APOE (dummy1)	0.538	0.853	0.106	0.448	0.414	0.328	0.965	0.162	0.622
	APOE (dummy2)	0.702	0.260	0.418	0.283	0.632	0.261	0.499	0.554	0.188
	FDG_SUVR_	0.022 *	<0.001 *	0.009 *	<0.001 *	0.002 *	<0.001 *	<0.001 *	0.105	<0.001 *
	FBB_SUVR_	0.649	0.056	0.040 *	0.162	0.061	0.382	0.378	0.017 *	0.079
R^2^		0.712	0.437	0.495	0.447	0.410	0.558	0.417	0.718	0.419
Stepwise ^†^										
	FDG_SUVR_	0.002	<0.001	0.001	<0.001	0.002	<0.001	<0.001	0.031	<0.001
	FBB_SUVR_	-	0.005	-	0.001	0.009	-	-	0.002	0.002
	Age	-	0.011	-	0.035	-	0.004	0.015	-	-
	EDU	-	-	-	-	0.011	-	0.005	-	-
R^2^		0.534	0.427	0.334	0.435	0.392	0.516	0.401	0.575	0.368
ADAS13 (*p*-value)										
		G12		G14		G16		G18		Total
Mode	EDU	≤12	>12	≤14	>14	≤16	>16	≤18	>18	
Enter										
	Age	0.601	0.001 *	0.753	0.002 *	0.300	0.007 *	0.016 *	0.166	0.004 *
	Sex (dummy)	0.724	0.516	0.489	0.706	0.347	0.852	0.461	0.862	0.634
	EDU	0.512	0.617	0.303	0.493	0.013^*^	0.735	0.023^*^	0.548	0.074
	APOE (dummy1)	0.785	0.648	0.216	0.335	0.614	0.379	0.684	0.148	0.907
	APOE (dummy2)	0.565	0.286	0.568	0.388	0.567	0.337	0.554	0.402	0.184
	FDG_SUVR_	0.015 *	<0.001 *	0.004 *	<0.001 *	<0.001 *	<0.001 *	<0.001 *	0.068	<0.001 *
	FBB_SUVR_	0.666	0.019 *	0.038 *	0.052	0.026 *	0.210	0.171	0.011 *	0.023 *
R^2^		0.753	0.506	0.542	0.515	0.509	0.559	0.485	0.759	0.487
Stepwise ^†^										
	FDG_SUVR_	<0.001	<0.001	0.002	<0.001	<0.001	<0.001	<0.001	0.028	<0.001
	FBB_SUVR_	-	<0.001	0.040	0.001	0.001	0.045	0.005	0.001	0.001
	Age	-	0.003	-	0.004	-	0.008	-	-	0.028
	EDU	-	-	-	-	0.007	-	-	-	-
R^2^		0.650	0.497	0.475	0.504	0.491	0.544	0.429	0.615	0.462
MMSE (*p*-value)										
		G12		G14		G16		G18		Total
Mode	EDU	≤12	>12	≤14	>14	≤16	>16	≤18	>18	
Enter										
	Age	0.187	0.174	0.448	0.266	0.839	0.551	0.771	0.031 *	0.432
	Sex (dummy)	0.472	0.787	0.976	0.611	0.721	0.726	0.755	0.407	0.941
	EDU	0.463	0.158	0.902	0.752	0.176	0.750	0.089	0.283	0.060
	APOE (dummy1)	0.768	0.896	0.204	0.623	0.454	0.552	0.579	0.950	0.734
	APOE (dummy2)	0.896	0.172	0.547	0.177	0.826	0.259	0.905	0.010	0.317
	FDG_SUVR_	0.532	<0.001 *	0.280	<0.001 *	0.017 *	<0.001 *	<0.001 *	0.113	<0.001 *
	FBB_SUVR_	0.713	0.138	0.033 *	0.786	0.055	0.669	0.061	0.672	0.092
R^2^		0.477	0.356	0.354	0.340	0.303	0.397	0.326	0.685	0.323
Stepwise ^†^										
	FDG_SUVR_	-	<0.001	-	<0.001	0.019	<0.001	<0.001	-	<0.001
	FBB_SUVR_	-	0.006	0.005	-	0.013	-	0.019	-	0.010
R^2^		0.325	0.328	0.244	0.308	0.263	0.362	0.300	-	0.294

Abbreviations: CDR, clinical dementia rating—sum of boxes; G12–18, groups with the 12-, 14-, 16-, 18-year cut-off educational attainment, accordingly; EDU, educational attainment in years; APOE, number of apolipoprotein E4 alleles; FDG_SUVR_, average standardized uptake value ratio (SUVR) of angular, temporal, and posterior cingulate in F-18 fluorodeoxyglucose positron emission tomography (PET); FBB_SUVR_, average SUVR of frontal cortex, anterior cingulate, precuneus cortex, and parietal cortex in F-18 florbetaben PET; ADAS11/13, Alzheimer’s disease assessment scale 11/13; MMSE, mini-mental state examination. * (*p* ≤ 0.05) indicates statistical significance. ^†^ In stepwise mode, only statistically significant independent variables with *p* ≤ 0.05 are presented.

## Data Availability

Not applicable.

## Supplementary Materials

The following supporting information can be downloaded at: https://www.mdpi.com/article/10.3390/medicina59050945/s1, Table S1: Comparisons in subgroups by the educational attainment for MCI participants; Table S2: Comparisons in subgroups by the educational attainment for AD participants; Table S3: Correlation analyses in subgroups by the educational attainment for MCI participants; Table S4: Correlation analyses in subgroups by the educational attainment for AD participants.
