# Supplementary material for: The Use of F-18 FDG PET-Based Cognitive Reserve to Evaluate Cognitive Decline in Alzheimer’s Disease, Independent of Educational Influence
Source: Medicina (Kaunas). 2023 May 14;59(5):945. doi: 10.3390/medicina59050945 (PMC10222488; doi:10.3390/medicina59050945)
Supplement: Supplementary file 1 [file medicina-59-00945-s001.zip › medicina-2330833-supplementary.pdf]

(*Supplementary material*)

Use of F-18 FDG PET-based cognitive reserve for evaluation of cognitive decline, independent of educational influence, in Alzheimer's disease

TABLE S1. Comparisons in subgroups by the educational attainment for MCI participants

		G12		G14		G16		G18	
EDU		≤12	>12	≤14	>14	≤16	>16	≤18	>18
Number		12	79	21	70	51	40	74	17
Age		71.1 ±5.5	71.9 ±8.2	70.8 ±7.1	72.1 ±8.1	70.0 ±8.3*	74.1 ±6.8*	71.1 ±7.7	75.0 ±8.0
Sex									
	M	8 (67%)	43 (54%)	12 (57%)	39 (56%)	26 (51%)	25 (63%)	40 (54%)	11 (65%)
	F	4 (33%)	36 (46%)	9 (43%)	31 (44%)	25 (49%)	15 (37%)	34 (46%)	6 (35%)
APOE†									
	0	5 (42%)	32 (41%)	10 (48%)	27 (39%)	23 (45%)	14 (35%)	30 (41%)	7 (41%)
	1	3 (25%)	23 (29%)	7 (33%)	19 (27%)	16 (31%)	10 (25%)	24 (32%)	2 (12%)
	2	1 (8%)	5 (6%)	1 (5%)	5 (7%)	3 (6%)	3 (8%)	5 (7%)	1 (6%)
CDR		1.9 ±1.1	1.5 ±1.0	1.5 ±1.0	1.6 ±1.0	1.5 ±.9	1.7 ±1.2	1.6 ±1.0	1.7 ±1.0
ADAS11		10.9 ±5.1	8.3 ±3.3	10.0 ±4.5	8.3 ±3.4	8.7 ±3.6	8.6 ±3.8	8.5 ±3.6	9.5 ±4.0
ADAS13		16.4 ±7.6	13.3 ±5.3	15.4 ±6.9	13.2 ±5.2	13.6 ±5.5	13.8 ±6.0	13.4 ±5.6	15.0 ±6.0
MMSE		27.1 ±2.1*	28.2 ±1.7*	27.3 ±1.9*	28.3 ±1.7*	27.7 ±1.9*	28.6 ±1.3*	28.0 ±1.8	28.5 ±1.2
FDG_{SUVR}		1.254 ±.065	1.241 ±.079	1.247 ±.066	1.242 ±.080	1.241 ±.083	1.245 ±.070	1.243 ±.076	1.243 ±.082
FBB_{SUVR}		1.204 ±.224	1.199 ±.257	1.196 ±.246	1.201 ±.255	1.194 ±.250	1.207 ±.257	1.119 ±.255	1.202 ±.245

Abbreviations: *MCI*, mild cognitive impairment; *G12-18*, groups with the 12-, 14-, 16-, 18-year cutoff educational attainment accordingly; *EDU*, educational attainment in year; *M*, male; *F*, female; *Dx*, diagnosis; *A*, Alzheimer's disease (AD), *I*, MCI, *N*, normal; *APOE*, number of apolipoprotein E4 allele; *CDR*, clinical dementia rating – sum of boxes; *ADAS11/13*, AD assessment scale 11/13; *MMSE*, mini-mental state examination; FDG_{SUVR}, average standardized uptake value ratio (SUVR) of angular, temporal, and posterior cingulate in ^{18}F-fluorodeoxyglucose positron

emission tomography (PET); FBB_{SUVR}, average SUVR of frontal cortex, anterior cingulate, precuneus cortex, and parietal cortex in ^{18}F-florbetaben PET

* These values were statistically significant with p-values ≤ .05 in the subgroup.

† APOE results were not available for all participants.

TABLE S2. Comparisons in subgroups by the educational attainment for AD participants

		G12		G14		G16		G18	
EDU		≤12	>12	≤14	>14	≤16	>16	≤18	>18
Number		3	27	10	20	15	15	26	4
Age		65.8 ±4.7	74.8 ±7.6	70.4 ±9.1	75.7 ±6.6	72.0 ±9.0	75.8 ±6.2	73.5 ±7.5	76.8 ±10.5
Sex									
	M	1 (33%)	18 (67%)	4 (40%)	15 (75%)	7 (47%)	12 (80%)	15 (58%)	4 (100%)
	F	2 (67%)	9 (33%)	6 (60%)	5 (25%)	8 (53%)	3 (20%)	11 (42%)	0 (0%)
APOE†									
	0	1 (33%)	3 (11%)	2 (20%)	2 (10%)	2 (13%)	2 (13%)	3 (12%)	1 (25%)
	1	0 (0%)	15 (56%)	3 (30%)	12 (60%)	7 (47%)	8 (53%)	15 (58%)	0 (0%)
	2	2 (67%)	7 (26%)	5 (50%)	4 (20%)	6 (40%)	3 (20%)	6 (23%)	3 (75%)
CDR		3.2 ±2.3	4.2 ±1.8	4.6 ±2.3	3.9 ±1.5	4.5 ±1.9	3.8 ±1.8	4.1 ±1.9	4.1 ±.8
ADAS11		22.4 ±4.4	18.9 ±5.6	21.7 ±7.3	18.0 ±4.1	21.3 ±6.1*	17.2 ±4.0*	19.3 ±5.4	18.8 ±7.2
ADAS13		33.4 ±4.4	29.4 ±7.3	32.6 ±9.2	28.4 ±5.5	32.0 ±7.8	27.5 ±5.6	29.9 ±6.7	28.8 ±10.6
MMSE		24.0 ±3.6	23.7 ±2.7	23.0 ±3.8	24.2 ±2.1	23.5 ±3.2	24.1 ±2.3	23.9 ±2.9	23.3 ±1.0
FDG_{SUVR}		1.164 ±.020	1.131 ±.117	1.114 ±.119	1.145 ±.108	1.114 ±.129	1.155 ±.090	1.130 ±.113	1.162 ±.111
FBB_{SUVR}		1.452 ±.396	1.486 ±.268	1.561 ±.224	1.443 ±.294	1.595 ±.217*	1.369 ±.285*	1.490 ±.275	1.432 ±.309

Abbreviations: AD, Alzheimer' s disease; *G12-18*, groups with the 12-, 14-, 16-, 18-year cutoff educational attainment accordingly; *EDU*, educational attainment in year; *M*, male; *F*, female; *Dx*, diagnosis; *A*, AD, *I*, mild cognitive impairment, *N*, normal; *APOE*, number of apolipoprotein E4 allele; *CDR*, clinical dementia rating – sum of boxes; *ADAS11/13*, AD assessment scale 11/13; *MMSE*, mini-mental state examination; FDG_{SUVR}, average standardized uptake value ratio (SUVR) of angular, temporal, and posterior cingulate in ^{18}F-fluorodeoxyglucose positron emission

tomography (PET); *FBB*$_{SUVR}$, average SUVR of frontal cortex, anterior cingulate, precuneus cortex, and parietal cortex in ^{18}F-florbetaben PET

* These values were statistically significant with p-values ≤ .05 in the subgroup.

† APOE results were not available for all participants.

TABLE S3. Correlation analyses in subgroups by the educational attainment for MCI participants

**CDR**									
	**G12**		**G14**		**G16**		**G18**		**Total**
**EDU (year)**	≤12	>12	≤14	>14	≤16	>16	≤18	>18	-
**Number**	12	79	21	70	51	40	74	17	91
**Age**†	-.076	.115	.043	.099	.117	.028	.082	.120	.093
**Sex**‡	-.026	-.033	-.073	-.038	-.007	-.089	-.053	.013	-.044
**EDU**†	-.188	.161	-.467*	.030	-.100	-.030	.001	.084	.021
**APOE**‡	.799*	-.045	.513*	-.097	.278	-.270	.071	-.163	.049
$\mathbf{FDG_{SUVR}}$†	-.634*	-.116	-.425	-.105	-.345*	.038	-.224	.099	-.164
$\mathbf{FBB_{SUVR}}$†	.666*	.022	.418	.009	.311*	-.120	.063	.253	.094
**ADAS11**									
	**G12**		**G14**		**G16**		**G18**		**Total**
**Age**†	.064	.186	.071	.088	.061	.313*	.148	.073	.151
**Sex**‡	-.410	-.110	-.398	-.101	-.209	-.168	-.235*	.101	-.176
**EDU**†	-.170	.044	-.305	.126	-.396*	.098	-.315*	-.088	-.151
**APOE**‡	.224	.063	.026	.133	.122	.049	.099	-.056	.101
$\mathbf{FDG_{SUVR}}$†	-616*	-.324*	-.446*	-.326*	-.259	-.442*	-.304*	-.456	-.333*
$\mathbf{FBB_{SUVR}}$†	.347	.232*	.175	.276*	.152	.341*	.139	.672*	.239*
**ADAS13**									
	**G12**		**G14**		**G16**		**G18**		**Total**
**Age**†	.170	.274*	.177	.291*	.153	.388*	.235*	.192	.242*
**Sex**‡	-.205	-.074	-.175	-.084	-.146	-.132	-.188	.176	-.115
**EDU**†	-.094	.034	-.248	.107	-.352*	.037	-.251*	-.205	-.115
**APOE**‡	.522	.113	.212	.157	.271	.004	.157	-.090	.165
$\mathbf{FDG_{SUVR}}$†	-.739*	-.412*	-.543*	-.422*	-.428*	-.444*	-.443*	-.400	-.431*
$\mathbf{FBB_{SUVR}}$†	.078	.325*	.279	.382*	.308*	.385*	.259*	.732*	.344*
**MMSE**									

	G12		G14		G16		G18		Total
**Age**†	.278	.012	-.091	.064	.003	-.100	.073	-.304	.046
**Sex**‡	.083	-.150	.024	-.161	-.133	-.016	-.133	.052	-.111
**EDU**†	-.140	.217	.041	.203	.159	-.053	.299*	-.273	.289*
**APOE**‡	.258	-.088	.071	-.113	-.154	.036	-.093	.187	-.069
**FDG_{SUVR}**†	.225	.349*	.351	.321*	.341*	.257	.340*	.157	.308*
**FBB_{SUVR}**†	-.330	-.151	-.332	-.129	-.198	-.170	-.202	.033	-.170

Abbreviations: *MCI*, mild cognitive impairment; *CDR*, clinical dementia rating - sum of boxes; *G12-18*, groups with the 12-, 14-, 16-, 18-year cutoff educational attainment accordingly; *EDU*, educational attainment in year; *APOE*, number of apolipoprotein E4 allele; *FDG_{SUVR}*, average standardized uptake value ratio (SUVR) of angular, temporal, and posterior cingulate in ^{18}F-fluorodeoxyglucose positron emission tomography (PET); *FBB_{SUVR}*, average SUVR of frontal cortex, anterior cingulate, precuneus cortex, and parietal cortex in ^{18}F-florbetaben PET; *ADAS11/13*, Alzheimer's disease assessment scale 11/13; *MMSE*, mini-mental state examination

† Age, EDU, FDG_{SUVR}, and FBB_{SUVR} represented the correlation coefficients of Pearson correlation analysis.

‡ Sex and APOE showed the correlation coefficients of Spearman correlation analysis.

* These values were statistically significant with p-values ≤ .05

TABLE S4. Correlation analyses in subgroups by the educational attainment for AD participants

CDR									
	G12		G14		G16		G18		Total
EDU (year)	≤12	>12	≤14	>14	≤16	>16	≤18	>18	-
Number	3	27	10	20	15	15	26	4	30
Age†	-.850	-.139	-.005	-.102	-.021	-.141	-.114	-.157	-.110
Sex‡	.000	-.041	.289	-.314	.235	-.505	-.037	-	-.048
EDU†	-	-.312	.211	-.084	.015	.018	-.191	-	-.152
APOE‡	-.866	.075	-.401	.218	-.457	.183	-.226	.544	-.103
FDG_{SUVR}†	.224	-.108	-.227	.020	-.177	.074	-.142	.533	-.110
FBB_{SUVR}†	-.966	-.173	-.141	-.436	-.116	-.610*	-.293	.143	-.263
ADAS11									
	G12		G14		G16		G18		Total
Age†	-.965	.056	-.149	.305	-.086	.342	-153	.420	-.051
Sex‡	.866	.131	.284	.000	.247	-.154	.234	-	.196
EDU†	-	-.282	-.125	-.147	-.152	.264	-.438*	-	-.335
APOE‡	-.866	-.082	-.382	-.025	-.447	.047	-.124	.258	-.099
FDG_{SUVR}†	-.560	-.252	-.137	-.267	-.130	-.262	-.226	-.234	-.228
FBB_{SUVR}†	-.849	.226	-.152	.213	-.154	.086	.053	.517	.126
ADAS13									
	G12		G14		G16		G18		Total
Age†	-.980	.003	-.119	.151	-.069	.126	-.187	.337	-.084
Sex‡	.000	.262	.142	.210	.217	.193	.337	-	-.126
EDU†	-	-.276	-.147	-.178	-.184	.100	-.412*	-	-.320
APOE‡	-.866	-.048	-.296	.026	-.328	.158	-.090	.258	-.054
FDG_{SUVR}†	-.130	-.292	-.156	-.325	-.165	-.332	-.252	-.347	-.267
FBB_{SUVR}†	-.996	.277	-.126	.299	-.092	.212	.105	.531	.182
MMSE									

	G12		G14		G16		G18		Total
Age†	.271	.193	-.025	.281	.011	.427	.187	.350	.171
Sex‡	.866	.051	.179	.268	.156	.158	.100	-	.114
EDU†	-	.157	-.031	-.205	.169	-.263	.189	-	.104
APOE‡	.000	-.048	.080	-.156	.106	-.198	.010	.272	.003
FDG_{SUVR}†	-.823	.123	-.156	.308	-.027	.312	.087	.855	.104
FBB_{SUVR}†	.537	-.193	-.064	-.065	-.018	-.106	-.109	-.094	-.099

Abbreviations: *AD*, Alzheimer's disease; *CDR*, clinical dementia rating - sum of boxes; *G12-18*, groups with the 12-, 14-, 16-, 18-year cutoff educational attainment accordingly; *EDU*, educational attainment in year; *APOE*, number of apolipoprotein E4 allele; *FDG_{SUVR}*, average standardized uptake value ratio (SUVR) of angular, temporal, and posterior cingulate in ^{18}F-fluorodeoxyglucose positron emission tomography (PET); *FBB_{SUVR}*, average SUVR of frontal cortex, anterior cingulate, precuneus cortex, and parietal cortex in ^{18}F-florbetaben PET; *ADAS11/13*, AD assessment scale 11/13; *MMSE*, mini-mental state examination

† Age, EDU, FDG_{SUVR}, and FBB_{SUVR} represented the correlation coefficients of Pearson correlation analysis.

‡ Sex and APOE showed the correlation coefficients of Spearman correlation analysis.

* These values were statistically significant with p-values ≤ .05
